# The Analysis of Medical Malpractice Litigation Related to Diagnosis of Headache in Japan: Mitigating Medicolegal Risks for Primary Care Physicians

**DOI:** 10.7759/cureus.89763

**Published:** 2025-08-10

**Authors:** Masayuki Ohira, Ryo Sumioka, Masaki Takao

**Affiliations:** 1 Department of General Internal Medicine and Clinical Laboratory, National Center of Neurology and Psychiatry Hospital, Tokyo, JPN; 2 Legal Department, Jinho Law Office, Tokyo, JPN

**Keywords:** diagnostic error, headache, japan, malpractice litigation, negligence

## Abstract

Background: Malpractice disputes are a major problem for physicians. Headache is one of the most common symptoms seen in medical settings, and therefore often an issue in malpractice cases. Little is known about the characteristics of headache in cases that end up as malpractice suits.

Methods: This study reviews all civil lawsuits involving headache that were identified in a Japanese database of lawsuits (Westlaw Japan). This study examined the basic characteristics, main issues, and results in each case, as well as the features of headaches, including clinical characteristics for secondary headaches and "red flags" identified by the court.

Results: In total, 2,906 cases were examined, of which 48 headache-related negligence lawsuits were retained for analysis. Judgments in these cases were delivered between January 2001 and November 2022. All 48 cases involved issues related to the diagnosis or treatment of headaches. Six cases were excluded because they were judgments from appeal courts or the Supreme Court, and judgments from lower courts in these cases were already included. Overall, 35 cases involved issues related to the diagnosis of headaches. No statistically significant differences were found between cases with and without negligence in terms of basic characteristics and the prevalence of red flags.

Conclusions: This study examined the basic characteristics of medical malpractice lawsuits related to the diagnosis and treatment of headache in Japan. No available features for avoiding medical malpractice and legal negligence were found. Even when no obvious red flags of secondary headache are identified, diagnostic errors could still lead to findings of negligence in malpractice claims.

## Introduction

The term “medical malpractice lawsuits” usually refers to civil cases in which patients or families seek monetary compensation for harms allegedly caused by failure to meet professional standards of care [1]. In general, the substantive content of law controlling medical malpractice lawsuits is similar in Japan, the United States, Canada, Western Europe, and Australia [2]. As in American tort law, the central elements of a malpractice claim in Japan brought under the Japanese Civil Code Article 709 are the establishment of a duty of care, breach of that duty, a causal link between the breach and the harm, and damages. The stress caused by medical malpractice claims can lead to anxiety and depression, and even burnout of medical professionals [3]. This could lead to a vicious circle of diagnostic errors and malpractice disputes [4,5]. It is therefore crucial for physicians to prevent or avoid medical malpractice litigation.

Theoretically, diagnostic errors, which are the result of perceptions and medical opinions, are not always the same as a breach of the duty of care owed to patients under a legal claim for negligence. Indeed, the initiation of malpractice suits has been reported to correlate poorly with actual findings of negligence, which were also not predictive of payments to the plaintiff [6]. A recent systematic review suggested that there was no association between measures of malpractice liability risk and healthcare quality and outcomes, and that greater tort liability was not associated with improved quality of care [7]. Not all clinical errors resulted in malpractice claims, and not all malpractice claims were the result of clinical errors [8].

This mismatch notwithstanding, diagnostic error is often related to claims for legal negligence in medical malpractice cases in Japan and other countries [9,10]. A few previous studies about Japanese medical malpractice litigation have included the diagnosis of headache [11,12]. However, to our knowledge, no study has examined the complexity of the relationship between details of diagnostic errors and legal negligence.

To explore this intricate relationship, we picked headache as an example. Headache is one of the most common symptoms seen in primary care settings. Its timely and correct diagnosis is sometimes challenging, and failure could lead to medical malpractice litigation [13]. Red flags, such as those included in the “SNNOOP10 list” of factors that may indicate a secondary cause of headache, are widely used to distinguish secondary from primary etiology in headaches [14]. This study aimed to examine the characteristics of medical malpractice litigation related to the diagnosis of headache in Japan. By identifying factors, especially clinical red flags, that may influence the findings of legal negligence, we aim to provide actionable insights for physicians to mitigate medicolegal risks in their routine practice. Its applicability may extend to other countries where laws controlling medical malpractice lawsuits are similar to those in Japan.

## Materials and methods

We conducted a retrospective review of medical malpractice cases involving the diagnosis and treatment of headaches. Cases were collected using Westlaw, a nationwide online database search engine for legal cases in Japan (https://www.westlawjapan.com). This is a comprehensive legal research engine that is used in various types of medical malpractice research in other countries. Westlaw Japan covers more than 300,000 cases in Japan dating back to the prewar era [15]. On August 5, 2024, we searched for cases using the keyword “headache” among civil cases classified as claims for damage with a judgment date after September 22, 2000, and involving proceedings in Japan. We chose September 22, 2000, as a limiting date because the Supreme Court in Japan delivered a landmark decision about causation in medical malpractice lawsuits on this date [16]. This judgment was a seminal decision about the cause-and-effect relationship between failure due to negligence and the death of a patient in medical malpractice. We also considered that it was necessary to put some limitations on the period of research to avoid old cases that were out of date and not suitable for current medical settings. The category of cases was limited to claims for damages, excluding cases such as administrative litigation and lawsuits related to inheritance and family matters. At this stage, all cases were screened by MO, who is certified as a fellow of the Japanese Society of Internal Medicine and is also a licensed Japanese attorney and a member of a law firm in Tokyo, Japan.

Next, we collected elements from court judgments, such as the date of the judgment, age and sex of the patient, final condition of patient including death or severe disability, the type of medical institution that was the defendant in each case (e.g., hospital or clinic), the court’s decisions about issues including diagnosis and treatment for headache, the final result of the judgment, such as dismissal or acceptance, and the final diagnosis associated with the headache in each case. The issues were classified into two categories: those related to diagnosis and those related to treatment for each case. The cases, including those related to the diagnosis of the headache, were divided into two groups based on the courts’ decisions, as follows: cases with and without negligence associated with the diagnosis. The basic features of each case were compared between the two groups.

Finally, to identify what characteristics of headache could lead to both misdiagnosis of secondary causes of headache and judgments of legal negligence about it, cases with issues related to diagnosis, known as “the SNNOOP10 list,” were scrutinized to identify any red flags for secondary headache [17]. This includes as follows: (1) systemic symptoms including fever, (2) a history of neoplasm, (3) any neurological deficit (including decreased consciousness), (4) sudden or abrupt onset of headaches, (5) being over 50 years old, (6) changes to the pattern or recent onset of new headaches, (7) positional headache, (8) precipitated by sneezing, coughing, or exercise, (9) papilledema, (10) progressive headache and atypical presentations, (11) pregnancy or puerperium, (12) painful eye with autonomic features, (13) post-traumatic onset of headache, (14) pathology of the immune system such as HIV, and (15) painkiller overuse or use of new drug at onset of headache. These features were decided based on the facts that each court recognized in its judgment rather than claims by plaintiffs or defendants. The frequency of red flags was compared between cases with and without negligence.

Standard descriptive statistics were used to summarize patient characteristics. Continuous variables were expressed as the median (interquartile range: 25-75%) and categorical variables as frequencies and percentages. The Mann-Whitney U-test was used to compare continuous variables between two independent groups. Fisher’s exact test was used to compare categorical variables. In all statistical analyses, a p<0.05 was considered to indicate statistical significance. All statistical analyses were performed using EZR, a graphical user interface for R (Tochigi, Japan: Jichi Medical University) [18]. Institutional review board approval was not required for this study because all the data were retrieved from public databases.

## Results

**Figure 1:**
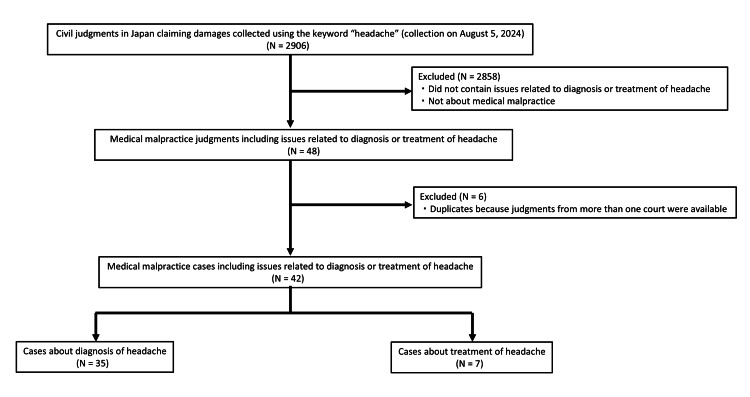
Study flowchart of the selection process for medical malpractice cases related to headache.

Overall, we found 2,906 civil cases claiming monetary damage using “headache” as the search term. All judgments were assessed by the first author (MO) to verify whether the main issues included diagnosis or treatment of headache. A total of 2,858 cases were excluded because they turned out to have no relation to this criterion. This left 48 cases covering legal issues related to the diagnosis or treatment of headaches. Of the 48 judgments, six were from appeal courts or the Supreme Court, and the lower courts’ judgments in these cases were already included in this study. We therefore analyzed 42 cases (Figure 1). Basic characteristics of these 42 cases are summarized in Table 1.

**Table 1:** Characteristics of cases in the study involving issues related to diagnosis or treatment of headache (n=42). ^a^Five cases were excluded because the age of the patient was unclear from the judgment. ^b^Some cases had multiple defendants. ^c^Some cases involved multiple specialists.

Characteristics	
Gender of patients	
Male, n (%)	20 (47.6)
Female, n (%)	21 (50.0)
Unknown, n (%)	1 (2.4)
Age in years, average^a^	43.0
Outcome for patients	
Death, n (%)	27 (64.3)
Sequelae, n (%)	15 (35.7)
Type of defendant^b^, n (%)	18 (18.0)
Hospital, n (%)	36 (85.7)
Clinic, n (%)	5 (11.9)
Doctor, n (%)	12 (28.6)
Specialty of doctors^c^,n (%)	
Neurosurgeon	12 (28.6)
Neurologist	5 (11.9)
Other	22 (52.4)
Unknown	6 (14.3)
Category of final diagnosis, n (%)	
Cerebral vascular disease	23 (54.8)
Brain tumor	5 (11.9)
Infectious disease	11 (26.2)
Others	3 (7.1)
Category of issue, n (%)	
Diagnosis	35 (83.3)
Treatment	7 (16.7)
Final result of cases, n (%)	
Acceptance	16 (38.1)
Dismissal	26 (61.9)

Overall, 27 patients (64.3%) died, and 15 (35.7%) were disabled to some extent. Neurosurgeons were the most common specialists involved in 12 cases (27.0%) followed by neurologists in five cases (11.0%). The final diagnosis was cerebrovascular disease in 23 cases (54.8%), infectious disease in 11 cases (26.2%), brain tumor in five cases (11.9%), and another condition in three cases (7.1%). The issues related to headache consisted of diagnosis, such as delay in diagnosis or misdiagnosis, in 35 cases (83%), and treatment, such as incorrect implementation of surgery or inappropriate control of postoperative patients, in seven cases (17%).

**Figure 2:**
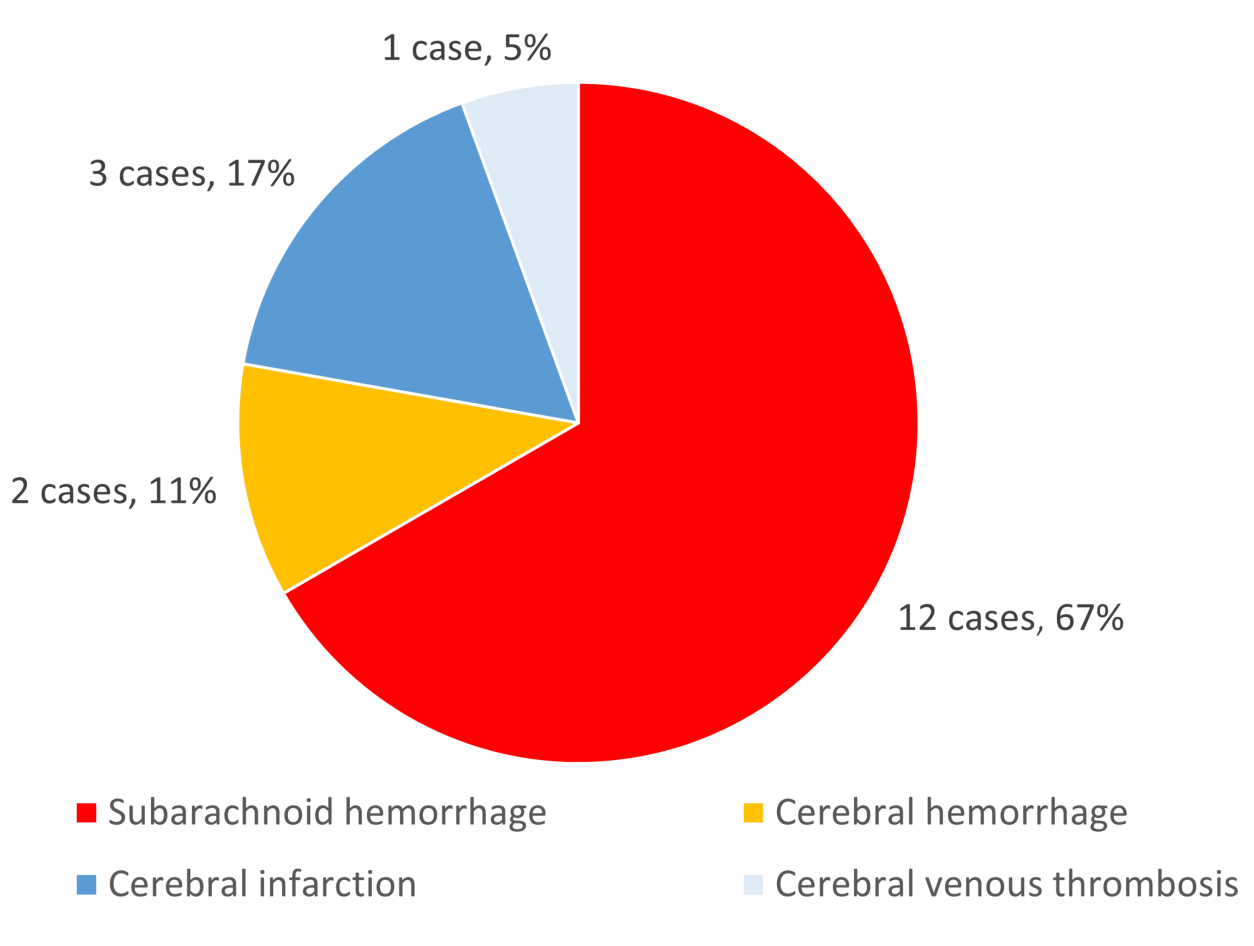
Final diagnosis in cases involving cerebral vascular disease included in the study (n, %).

The features of 35 cases, including issues related to the diagnosis of headache, were classified into two groups by whether legal negligence was found, and are summarized in Table 2. Overall, there were 12 cases where negligence was identified and 23 without negligence. The final diagnosis in all cases discussing diagnostic errors was a secondary cause of the headache. Subarachnoid hemorrhage was most common in cases of cerebrovascular disease (Figure 2).

**Table 2:** Comparison of parties’ characteristics between cases with and without negligence in this study (n=35). ^a^One case was excluded because exact sex of patient was not included in the judgment. ^b^Four cases were excluded because exact age of patients was not included in the judgment. ^c^Five cases were excluded because exact specialty of doctors was not included in the judgment. ^d^Fisher’s exact test. ^e^Mann-Whitney U test.

Characteristics	Cases with negligence (n=12)	Cases without negligence (n=23)	Odds ratio (95% confidence interval) or statistic value for Mann-Whitney U test	p-Value
Male, n (%)^a^	7 (58.3)	8 (36.4)	2.38 (0.47-13.2)	0.29^d^
Female, n (%)	5 (41.7)	14 (63.6)	-	-
Age in years, median (IQR)^b^	33.5 (27.3-51.3)	43.0 (35.0-59.5)	70.0	0.08^e^
Type of defendant, n (%)				
Hospital	10 (83.3)	21 (91.3)	0.49 (0.03-7.63)	0.59^d^
Clinic	2 (16.7)	2 (8.7)	-	-
Specialty of doctors other than neurologist and neurosurgeon, n (%)^c^	6 (54.5)	5 (26.3)	0.31 (0.05-1.84)	0.24^d^

A total of 11 infectious disease cases included five people with meningitis, three with cerebritis, two with brain abscess, and one with endocarditis. There were no statistical differences between the basic characteristics of the two groups (Table 2). The red flags for secondary headache were analyzed in these 35 cases. The frequency of occurrence was compared between cases with and without legal negligence (Table 3).

**Table 3:** Comparison of characteristics of headache between cases with and without negligence (n=35). ^a^Six cases of other categorical diseases were excluded because the numbers were too small for analysis. ^b^Fisher’s exact test.

Characteristics	Cases with negligence (n=12)	Cases without negligence (n=23)	Odds ratio (95% confidence interval)	p-Value
Category of final diagnosis^a^				
Cerebral vascular disease, n (%)	5 (50.0)	13 (68.4)	0.47 (0.07-2.94)	0.43^b^
Infectious disease, n (%)	5 (50.0)	6 (31.6)	-	-
Red flags, n (%)				
Systemic symptoms including fever	6 (50.0)	8 (34.8)	1.84 (1.36-9.70)	0.48^b^
Neurological deficit or dysfunction	7 (58.3)	10 (43.5)	1.79 (0.36-9.58)	0.49^b^
Sudden or abrupt onset of headache	3 (25.0)	7 (30.4)	0.76 (0.10-4.52)	1.00^b^
Over 50 years old	4 (33.3)	8 (34.8)	0.93 (0.16-5.01)	1.00^b^
Pregnancy or puerperium	1 (8.3)	3 (13.0)	0.61 (0.01-8.76)	1.00^b^

Only fever, neurological deficit or dysfunction, sudden or abrupt onset of headache, being over 50 years old, and pregnancy or puerperium were compared, and no statistical differences were found in these categories. The other factors on the SNNOOP10 list, such as neoplasm history and papilledema, were not analyzed because there were fewer than two cases of each.

## Discussion

In this study, all cases of medical malpractice litigation in Japan involving the diagnosis of headache had secondary causes of the headache, such as subarachnoid hemorrhage and meningitis. There were no statistical differences in characteristics or red flags between cases with and without legal negligence. This suggests that no feature of headache, including the red flags in the SNNOOP10 list, could be used as a way to avoid findings of legal negligence in Japan. Theoretically, diagnostic errors made when fewer red flags were present should indicate that physicians are less likely to be held responsible. Similarly, physicians who see cases with more red flags might be expected to suspect secondary causes of headache and should therefore be more likely to be found legally responsible. However, this was not the case in practice.

We found that all the headaches reported in cases with legal issues about diagnostic errors had secondary causes. These findings are in line with previous reports [19,20]. In general, headache is known to have a risk of misdiagnosis [21]. It is therefore essential for physicians to rule out life-threatening secondary causes of headaches, such as subarachnoid hemorrhage or meningitis [22]. This action of confirming or excluding these secondary causes of headaches is considered critical for mitigating medicolegal risks and avoiding medical malpractice litigation in Japan and elsewhere [13].

No features, including the red flags in the SNNOOP10 list, showed statistically significant differences in prevalence between cases with and without negligence (Tables 2, 3). A younger age was relatively common in cases involving legal negligence in this study. However, older age is included in the SNNOOP10 list. These findings may seem contradictory to previous reports examining these red flags in cases of diagnosis of secondary headache [14]. A previous study using the SNNOOP10 list reported that the group experiencing headaches from secondary causes was significantly older than the group with primary causes of headaches in emergency departments in Japan [23]. This could be one reason for the inconsistency between diagnostic error and legal negligence. Other red flags on the SNNOOP10 list were not found in cases with negligence. These results could imply that legal negligence was affected by various factors other than simple medical standards and opinions. It may therefore be appropriate to distinguish between legal negligence and diagnostic errors in medical research about medical malpractice litigation, which previous studies have not done [11,24,25].

In this study, we did not include the outcome of lawsuits, compensatory damages, and payments for pain and suffering. Some studies on medical malpractice lawsuits in Japan have reported these numbers [11,26]. However, unlike US tort litigation, this type of analysis has little meaning in medical research. First, the outcomes of each case (i.e., acceptance or dismissal) are not guaranteed to be medically accurate. They could be affected by the legal strategy used by either party [6]. Second, damages and payments in medical malpractice litigation cases in Japan closely follow those for personal injuries resulting from automobile accidents. These damages are determined by reference to what is colloquially known as the “Red Book,” a guideline for the calculation of damages following traffic accidents that is published annually by one of the lawyers’ associations in Tokyo. Damages in malpractice litigation in Japan are therefore calculated on the basis of fixed factors, such as age, gender, and wage-earning status of those involved and the costs of hospitalization, rather than whether negligence occurred.

This study had several limitations because of the legal system in Japan. First, the only available material for this kind of study is judgments from medical malpractice litigation in Japan. For example, we were unable to use data on medical malpractice lawsuits held by insurance companies. Other claims and types of information are not publicly available. Second, medical malpractice litigation statistics suggest that about half of the cases were settled by reconciliation, and their results are therefore not publicly available [27]. Consequently, we were unable to include these claims in this study. Third, details of some cases ending in a judgment are also not publicly available. These factors limited the number of cases included in this study.

## Conclusions

This study examined the basic characteristics of medical malpractice lawsuits related to the diagnosis and treatment of headache in Japan. It highlights that simply focusing on medical "red flags" may not be sufficient to mitigate medicolegal risks in Japan. Physicians should always consider potential secondary causes of headache regardless of the presence or absence of red flags, to help them avoid medical malpractice disputes or diagnostic errors.
